# Non-invasive prenatal testing of beta-hemoglobinopathies using next generation sequencing, *in-silico* sequence size selection, and haplotyping

**DOI:** 10.3325/cmj.2024.65.180

**Published:** 2024-06

**Authors:** Henry A. Erlich, Lily Ko, Jiyae Lee, Katrina Eaton, Cassandra D. Calloway, Ashutosh Lal, Reena Das, Manu Jamwal, Christian Lopez-Pena, Steven J. Mack

**Affiliations:** 1 UCSF Benioff Children’s Hospital Oakland Research Institute, Oakland, CA, USA; 2 Department of Pediatrics, University of California, San Francisco, Oakland, CA, USA; 3 Post-Graduate Institute of Medical Education and Research, Chandigarh, India

## Abstract

**Aim:**

To develop a non-invasive prenatal test for beta-hemoglobinopathies based on analyzing maternal plasma by using next generation sequencing.

**Methods:**

We applied next generation sequencing (NGS) of maternal plasma to the non-invasive prenatal testing (NIPT) of autosomal recessive diseases, sickle cell disease and beta-thalassemia. Using the Illumina MiSeq, we sequenced plasma libraries obtained via a Twist Bioscience probe capture panel covering 4 Kb of chromosome 11, including the beta-globin (*HBB*) gene and >450 genomic single-nucleotide polymorphisms (SNPs) used to estimate the fetal fraction (FF). The FF is estimated by counting paternally transmitted allelic sequence reads present in the plasma but absent in the mother. We inferred fetal beta-globin genotypes by comparing the observed mutation (Mut) and reference (Ref) read ratios to those expected for the three possible fetal genotypes (Mut/Mut; Mut/Ref; Ref/Ref), based on the FF.

**Results:**

We bioinformatically enriched the FF by excluding reads over a specified length via *in-silico* size selection (ISS), favoring the shorter fetal reads, which increased fetal genotype prediction accuracy. Finally, we determined the parental *HBB* haplotypes, which allowed us to use the read ratios observed at linked SNPs to help predict the fetal genotype at the mutation site(s). We determined *HBB* haplotypes via Oxford Nanopore MinION sequencing of a 2.2 kb amplicon and aligned these sequences using Soft Genetics’ NextGENe LR software.

**Conclusion:**

The combined use of ISS and *HBB* haplotypes enabled us to correctly predict fetal genotypes in cases where the prediction based on variant read ratios alone was incorrect.

Beta-hemoglobinopathies, sickle cell disease (SCD) and beta-thalassemia (β-Thal), are autosomal-recessive monogenic diseases that result from mutations in the beta-globin (*HBB*) gene, and are the most common monogenic diseases in the world. SCD results from homozygosity for the sickle hemoglobin variant, a Glu6Val missense mutation (rs334) in the *HBB* gene (1), while β-Thal results from *HBB* mutations that reduce or prevent beta-globin synthesis (2,3). SCD is caused by a single variant, while β-Thal is caused by many single-nucleotide, poly-nucleotide, frameshift, and deletion mutations (4).

Prenatal testing for these autosomal recessive diseases by using amniocentesis and chorionic villus sampling has been standard clinical practice for decades but it confers some risk to the fetus (5,6). A non-invasive test for these diseases would have significant clinical benefits (7).

Following the discovery of fetal DNA in the maternal plasma (8) and the implementation of next generation sequencing (NGS) and digital polymerase chain reaction (PCR), the development of non-invasive prenatal testing (NIPT) based on the analysis of plasma libraries has become well-established for chromosomal aneuploidies (9,10). The clonal property of NGS allows the quantitative resolution of mixtures, such as that of the fetal and maternal DNA in plasma, by sequencing the components separately. Recently, we and others have demonstrated that NGS analysis of plasma libraries can also be used for the more challenging diagnosis of autosomal recessive diseases, such as hemoglobinopathies (11-14). In this approach, the fetal genotype is predicted by counting Illumina MiSeq sequence reads from plasma libraries and comparing the observed ratios of disease-causing mutation (Mut) alleles and reference (Ref) alleles to those expected for the three possible genotypes (Mut/Mut, Mut/Ref, and Ref/Ref). The expected ratios for the three possible genotypes are based on estimates of the fetal fraction (FF), which is determined by counting allelic sequence reads present in the plasma and absent in the mother.

This strategy for fetal genotype prediction has two limitations. First, when the FF is low, the expected ratios for the three possible genotypes are not far apart, and the statistical confidence in the predicted fetal genotype is correspondingly lower. Second, the prediction is based on the observed sequence read ratios at only one or two positions, which limits the statistical confidence. To specifically address the limitations of NIPT for hemoglobinopathies using individual SNPs, we developed a strategy that 1) bioinformatically increases the FF and 2) uses parental *HBB* haplotypes to incorporate the allelic read ratios observed at linked SNPs in our prediction at the mutation site(s).

The combined use of ISS to increase the fetal fraction and haplotyping of the parental DNA to consider the read ratios at linked SNPs provides increased confidence in the fetal genotype prediction. This strategy is critical to the development of a robust, non-invasive prenatal test for autosomal recessive diseases, such as beta-hemoglobinopathies.

## Methods

The probe design, specimen collection, and probe capture/NGS methods applied in this work were performed as previously described by Erlich et al (11). DNA library preparation, probe capture enrichment, and sequencing methodologies were modified as described below.

### Participants

Maternal and paternal whole blood and chorionic villus samples (CVS) for families in which both parents were known to carry *HBB* mutations were collected at the Post-Graduate Institute of Medical Education and Research in Chandigarh, India (114 families), and at the University of California, San Francisco (UCSF) Benioff Children’s Hospital Oakland, in Oakland, CA, US (7 families).

This project was approved by the Institutional Review Board of the Children’s Hospital Oakland Research Institute, the UCSF Human Research Protection Program, the ethical collaborative committee of the Post Graduate Institute of Medical and Educational Research, and the Health Ministry Screening Committee of the Indian government for out-of-country sample transfer.

### DNA library preparation

We previously (11) sheared DNA extracted from whole blood and plasma to 250 bp before Illumina MiSeq sequencing. For the work described here, extracted plasma DNA was not sheared prior to sequencing. Given the mean fragment size of plasma DNA, between 145 and 201 bp (15), shearing to 250 bp was not deemed necessary.

As described below, DNA was extracted from whole blood, PCR-amplified, and sequenced by using Oxford Nanopore Technologies’ (ONT) (Oxford, UK) MinION platform. For ONT sequencing, a library for single-individual sequencing runs was prepared by using ONT Ligation Sequencing kits (SQK-LSK109), and a multiplex library was prepared by using Ligation Sequencing gDNA Native Barcoding kits (SQK-NBD114.96) according to the manufacturer’s specifications.

### Probe capture enrichment

Our previously described Probe Capture protocol (11,16) was modified to use a custom Twist Bioscience (South San Francisco, CA, USA) probe panel (PanHeme panel TE-95715506). This panel covers 4 kb of chromosome 11, and additionally includes 436 genomic (non-*HBB*) SNPs. This protocol involved 11 cycles of PCR amplification following adapter ligation and 15 cycles following probe capture.

### Genomic DNA PCR amplification and quantification

To sequence the entire *HBB* gene and flanking SNPs, we amplified a 2252 nucleotide-long segment by using primers A and D described by Chan et al (17). PCR amplification was performed by using 25 uL AmpliTaq Gold™ 360 Master Mix (1X, Applied Biosystems™, Waltham, MA, USA), 1 uL of primers (0.2 uM for each primer), and 150 ng of genomic DNA in PCR-grade water in 50 uL reaction volumes. After 10-minute denaturation at 95 °C, 33 PCR cycles (15 seconds at 95 °C, 30 seconds at 58.5 °C, and 2 minutes at 72 °C) were followed by 7 minutes of extension at 72 °C. PCR products were quantified via PicoGreen, by using the Quant-iT™ PicoGreen dsDNA™ Assay kits (Invitrogen, Waltham, MA, USA), and BioAnalyzer, by using Agilent DNA 7500 kits (Agilent, Santa Clara, CA, USA).

### DNA sequencing

Plasma DNA was sequenced on the Illumina MiSeq platform as previously described (11). Some plasma libraries were sequenced with the MiSeq v3 2 × 600-cycle reagent kits, and others were sequenced with the MiSeq v2 300-cycle reagent kits, run for either 150 or 175 cycles, given the short DNA fragments present in the plasma.

The 2.2 kb PCR product containing the *HBB* gene was PCR-amplified from DNA extracted from parental whole blood and CVS specimens, and sequenced by using the ONT MinION system. Singleplex sequencing was performed on a MinION Mk1b instrument by using R.9.4.1 and R10.4.1 flowcells, with MinKNOW software (version 5.0.5) over 4 hours. Multiplex sequencing was performed by using the MinION R10.4.1 flowcells, with MinKNOW software (version 5.0.5) over 3.5 hours.

### Data analysis and informatics

MiSeq read processing, elimination of PCR duplicates (11) (deduplication or dedup), and ISS were performed with the *size_selection_and_deduplication.py* script, available at *github.com/kaeaton/NIPT_Informatics*, which controls the *Fastp* (18), *BWA-MEM* (19), *Genome Analysis Toolkit* 4 (20) (GATK), and Gencore (21) command line applications (described below). Zipped, MiSeq-generated, fastq-formatted paired end reads are first processed by *Fastp*, which trims any remaining MiSeq adapter sequences, manages unique molecular identifier (UMI) (22) sequences, performs overlap analysis of paired-end reads, replaces mismatched, low-quality nucleotides, and performs ISS when desired, through application of the user-specified ‘–length limit’ parameter. The end product of the *Fastp* process is paired ‘R1’ and ‘R2’ fastq read files.

The fastq-formatted reads were aligned to the GRCh37/hg19 reference with the Burrows-Wheeler Alignment (BWA) tool, implemented as BWA MEM, which returns a SAM file. GATK converted this into a BAM file, which is inspected by GATK’s FixMateInformation tool, ensuring position matches within paired reads. Pair reads were then sorted by genomic coordinates by the GATK SortSAM tool before removal of PCR duplicates, based on the UMI tags.

PCR duplicates (dedup) were removed by Gencore, which builds consensus sequences by using the duplicate reads, providing the longest, most accurate consensus read per UMI. The default minimum number of reads (n = 1) is used to build consensus sequences, to ensure that all probed regions are included.

The resulting plasma reads were aligned and analyzed with Soft Genetics’ NextGENe software (State College, PA, USA) (23) version 2.4.2.2 by using GRCh37/hg19 genomic coordinates and previously described settings (11). Aligned reads were post-processed for variant calling, identifying *HBB* SNPs.

SoftGenetics’ NextGENe LR software (version 1.0.4.3) was used to analyze MinION singleplex and multiplex FASTQ data files, identifying *HBB* SNP haplotypes for maternal, paternal, and CVS specimens. In particular, NextGENe LR can phase *HBB* SNPs with the common 619 bp intron 2 – exon 3 deletion variant (GRCh37/hg19 chr11: 5246486-5247107) (24,25). The average read depth per ONT sequencing run was ~ 1 million reads, so that the read depth for multiplexed sequence runs was ~ 100 000 reads per specimen.

Sequence reads were aligned to the GRCh37/hg19 human genome reference sequence positions chr11:5246155-5248406, with 75% homology. NextGene LR indel percentage and variant effective percentage thresholds were set at 30% and 10%, respectively. Given the error rate of MinION sequencing, variant allele calls were made for minor allele sequence read frequencies >16%. As *MinKNOW* generates multiple ~ 10MB FASTQ files for each subject/barcode, all FASTQ files for a given subject/barcode were loaded into *NextGENe LR* and analyzed as a “single sample.”

## Results

To increase the accuracy of predicting the fetal genotype from sequence read ratios from the plasma libraries, we 1) bioinformatically increased the fetal fraction of plasma reads by ISS and 2) determined the beta-globin haplotypes by Oxford Nanopore sequencing of parental blood samples.

### In-silico size selection

The length distribution of Illumina MiSeq sequence reads from fetal DNA was compared with maternal reads for all informative SNPs in the plasma library. Informative SNPs are those for which an allele detected in the plasma is absent in the mother, and therefore assumed to have been transmitted from the father to the fetus. For example, if the mother is A/A, and 5% of the plasma reads are T, we can compare the length of fetal T reads with the length of the predominantly maternal A reads (we assume that 5% of the plasma A reads are from the fetus). Since half of the fetal reads will be A, the proportion of maternal A reads is 100% minus (FF%/2). Since the fetal reads (eg, T) tended to be slightly shorter than the predominantly maternal reads (eg, A), the FF could, in principle, be bioinformatically increased by excluding the longer sequence reads.

**Figure 1:**
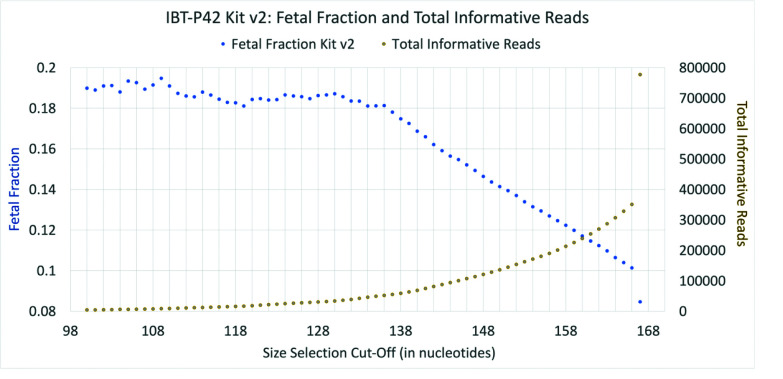
*In-silico* size selection cut-off values. The IBT-42 plasma library was sequenced on an Illumina MiSeq using a v2 2 × 300 kit run for 175 cycles. Read alignment, polymerase chain reaction duplicate removal, and *in-silico* size selection were applied.

This comparison was initially performed for all informative SNPs in a plasma library sequenced with Illumina MiSeq v3 600 cycle kits, as previously described (11). Figure 1 shows the analysis with a v2 2 × 300-cycle kit run for 175 cycles. The FF is estimated by counting the obligate fetal reads in a plasma library, calculating the average fetal proportion over all informative SNPs, and then multiplying by two. To systematically monitor the increase in the FF achieved by bioinformatically excluding sequence reads above a defined length, we excluded reads sequentially starting from 167 bp (Figure 1). As shown, the FF increased, peaking at around 135 bp. The number of informative reads for calculating the FF is shown on the right y-axis. These are reads for SNPs where the mother is homozygous. From a statistical perspective, the increase in FF needs to be balanced against the reduction in read count and the resulting increase in noise. The reads informative for the most challenging fetal genotype prediction are derived from the SNPs where the mother is heterozygous. Predicting the fetal genotype for SNPs where the mother is homozygous is easier.

**Figure 2:**
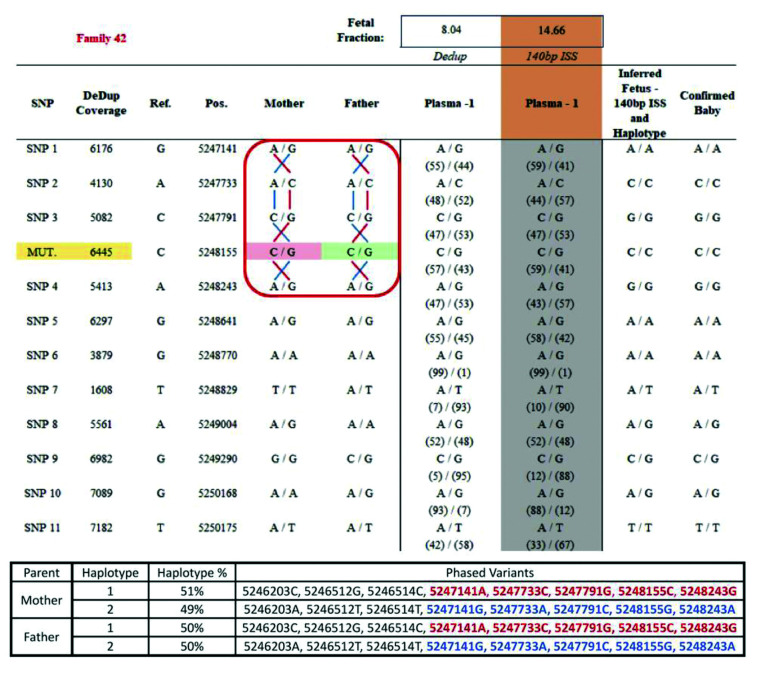
*In-silico* size selection (ISS) and haplotype data increase the confidence and accuracy of fetal genotype prediction. Inferred fetal genotypes for family IBT-42 based on plasma read ratios, ISS, and *HBB* haplotypes, are shown in the upper panel. For each of the 12 single nucleotide polymorphisms (SNP) detected in maternal plasma, the post-deduplication read-depth (dedup coverage), hg19 reference sequence (ref.), hg19 chromosome 11 reference coordinate (pos), maternal and paternal genotype, plasma ratios (plasma-1), inferred fetal genotype (inferred fetus -140 bp ISS and haplotype), and the fetal genotype confirmed via ON MinION sequencing of the chorionic villus samples (confirmed baby) are shown. In addition, the fetal fraction (FF) after deduplication and after ISS at 140 bp is shown at the top for both plasma ratios. The shaded plasma ratios were used to make the prediction. The *HBB* disease-causing SNP (*NC_000011.9:g.5248155C>G*) is highlighted. The phase between SNPs, as determined by ONT MinION sequencing, is shown for SNPs 1-4 and Mut in the red box, with variants on the same chromosome connected via red or blue lines. Phased SNP variants derived from ONT MinION sequencing of the 2.2 KB *HBB* amplicon for both parents are shown in the lower panel. The fraction of pertinent reads is shown (haplotype %) for each haplotype. Each SNP variant detected is shown in phase with its neighbors (phased variants) on the same line, with SNPs that were present among the fetal genotype reads presented in red or blue.

The goal of bioinformatically increasing the FF by ISS is to increase the accuracy and statistical confidence in the fetal genotype predictions based on comparing the observed and expected sequence read ratios in the plasma library. Increasing the FF will change the expected read ratios and, with the exception of a heterozygous fetus, can alter the observed read ratios as well. Figure 2 illustrates a case in which ISS altered the plasma read ratios, leading to more accurate fetal genotype predictions. As illustrated for seven families in Table 1, our application of ISS to increase the FF can help correct fetal genotype predictions, based solely on mutation site read ratios.

**Table 1:** Ratios and predictions of clinically significant mutations across seven families accomplished using *in-silico* size selection (ISS) and haplotyping

Family	Genomic position (hg19)	Parental genome	Variant ratio*	Predicted^†^ genotype	Fetal fraction (%)	Ratio after *in-silico* size selection	Prediction after *in-silico* size selection	Fetal fraction after *in-silico* size selection	Haplotype-based prediction	Baby's confirmed genotype
PST 5**^‡^**	5246959	Maternal	53G/47T	**G/G^¶^**	6.46	51G/49T	G/T	7.41 (190 bp)		G/T
	5248234	Paternal	4Del/96A	Del/A		4Del/96A	Del/A			Del/A
	5248235	Paternal	4Del/96G	Del/G		4Del/96G	Del/G			Del/G
IBT 31**^‡§^**	5248301	Maternal	48G/52T	**G/T or T/T**	6.81	49G/51T	**G/T**	4.92 (160 bp)	T/T	T/T
	5248225	Paternal	1Ins /100C	**Ins/C or C/C**		2Ins/100C	Ins/C			Ins/C
IBT 43**^‡§^**	5248155	Maternal	56C/44G	**C/C**	22.9	59C/41G	**C/C**	26.5 (160 bp)	C/G	C/G
	5248219	Paternal	93G/7T	G/T		88G/12T	G/T		G/T	G/T
IBT 29^§^		Maternal		619 Deletion	12.2				619 Deletion	619 Deletion
	5247993-5247995	Paternal	2Del/98A	**Del/A**					A/A	A/A
IBT 53**^‡§^**	5248219	Maternal/Paternal	54G /46T	**GT/ or G/G**	8.27	58G/43T	G/G	15.24 (140 bp)	G/G	G/G
IBT 63**^‡§^**	5248389	Maternal	47A/53G	**A/G or G/G**	6.27	58A/42G	A/G	12.91 (140 bp)	A/G	A/G
	5248225	Paternal	2Ins/100C	Ins/C		2Ins/100C	Ins/C		Ins/C	Ins/C
IBT 69**^‡^**	5238389	Maternal	47A /53G	**A/G or G/G**	8.12	47A/53G	A/G	14.09 (140 bp)	**A/G or G/G**	A/G
	5247976	Paternal	3Ins /100A	Ins/A		3Ins/100A	Ins/A		Ins/A	Ins/A

*For each position, the plasma variant ratio identifies the percentage of reads with the specific variants. For example, the 53G/47T ratio for position 5246959 indicates that 53% of the reads have a G sequence at this position, while 47% have a T sequence.

**†**Fetal genotypes at the mutation sites were predicted based on plasma ratio and FF values.

‡ISS was performed for these six families with size limits at 190 bp, 160 bp, and 140 bp to enrich the FF. The 190 bp cutoff was applied for MiSeq runs performed with the v3 kit for 300 cycles, the 160 bp cutoff was for runs performed with the v2 kit for 175 cycles, and the 140 bp cutoff was for runs performed with the v2 kit for 150 cycles.

§Haplotypes were determined by using SoftGenetics’ NextGENeLR software after base calling via the Oxford Nanopore Technologies’ MinKNOW platform.

¶Incorrect predictions are highlighted in bold.

### HBB haplotype analysis

The application of parental SNP haplotype information, in concert with the plasma sequence read ratios observed at SNPs linked to the mutation site(s), could increase the accuracy and statistical confidence in fetal genotype predictions. We adopted an *HBB* haplotype strategy using ONT MinION long read sequencing, to sequence a 2.2 kb *HBB* region amplified from parental DNA. NextGENe LR (v1.0.4.3) identifies the two parental haplotypes for each parent from the FASTQ, typically using ~ 1M reads per singleplex subject and ~ 75K reads per multiplex subject. NextGENE LR can phase SNPs with the common 619 deletion, allowing the use of linked SNPs alleles to predict whether this deletion was transmitted to the fetus (Table 1).

Figure 2 illustrates the combined use of ISS and *HBB* haplotype analysis to incorporate sequence read ratio data at linked SNPs into the mutation site prediction for family IBT-42. ISS increased the FF from 8.04 to 14.66. For SNP1, the observed read ratio changed from 55A/44G to 59A/41G, making the correct prediction of an A/A fetal genotype more likely. For SNP2, the ratio changed from 48A/52C to 44A/57C, making the correct prediction of a C/C genotype more likely. For SNP3, however, the ratios both pre- and post-ISS were 47C/53G, consistent with either a C/G heterozygote or a G/G homozygote. The haplotype information is required to correctly predict the G/G fetal genotype. At the mutation site, the C/C was correctly predicted by the 57C/43G ratio alone, but the ISS and haplotype analyses provided additional confidence in the prediction. For SNP4, the observed 47A/53G was consistent with either an A/G or a GG fetal genotype, but the ISS (43A/57G) and the haplotype analysis predicted the correct G/G genotype.

Table 1 illustrates how the ISS and haplotype analyses can help predict fetal genotype at the clinical mutation sites of seven additional families, including a family carrying the 619bp deletion. In some cases, maternal and paternal genomes displayed mutations at different positions. Families for which incorrect predictions were initially made on the basis of plasma ratios are shown. ISS and haplotype information were used to make correct predictions in these families. Incorrect predictions are highlighted in bold. The predicted genotype is the result returned by our algorithm, based solely on the observed and expected variant ratios. The expected ratios for each possible genotype are based on the fetal fraction (FF). The FF identifies the percent of plasma reads that is derived from the fetus, averaged over all informative SNPs.

For PST 5 at position 5246969, the plasma ratio after 190 bp ISS indicated that the G/T genotype was the most probable, resulting in a correct prediction. For IBT 31, 160 bp ISS provided higher confidence to predict Ins/C genotype at the paternal mutation site. However, the prediction based on the observed ratio at the maternal mutation site after ISS changed from inconclusive (G/T or T/T) to incorrect (G/T). The haplotype analysis indicated the correct T/T genotype. This case was anomalous in that the ISS did not increase the FF. For IBT 43, 160 bp ISS provided higher confidence to predict the G/T genotype at the paternal mutation site. However, the prediction at the maternal mutation site was still incorrect after ISS. For IBT 53, haplotype analysis predicted the correct C/G genotype, but 140 bp ISS provided higher confidence to predict the G/G genotype at both parental mutation sites. For IBT 63, the paternal genotype at position 5248389 was G/G (data not shown), and therefore fetal genotype was predicted to be A/G, despite the 58A/42G ratio observed after ISS. The haplotype analysis confirmed the A/G genotype. For IBT 69, although the observed ratios were the same before and after 140 bp ISS, the expected ratios changed so that ISS provided higher confidence to predict the correct A/G genotype at the maternal mutation site. The observed ratios at the linked SNPs were not sufficient to infer which parental haplotype was transmitted to the fetus so, in this case, the haplotype information could not aid in the fetal genotype prediction. For IBT 29, the prediction of the paternal mutation based on the plasma ratio and fetal fraction was incorrect. The application of ONT haplotypes changed the prediction from incorrect (Del/A) to correct (A/A).

## Discussion

The strategy of inferring fetal genotypes by comparing the observed ratios of allelic sequence reads to the expected ratios for the possible genotypes is limited by the FF and by considering the observed read ratios at only the mutation site. We have addressed these limitations by bioinformatically increasing the FF by ISS and using parental haplotyping to incorporate data from linked SNPs.

Based on the observation that fetal DNA may be slightly shorter than maternal DNA in plasma (26), we and others have shown that it is possible to bioinformatically increase the FF by excluding longer sequence reads, via ISS (11,14). Here, we demonstrated how the length distributions of fetal reads are skewed to shorter lengths relative to maternal sequence reads, and how the FF increases as a function of the excluded read length.

In our previous work, we used the availability of either an informative sibling or short-range haplotypes determined from overlapping MiSeq reads to aid in the prediction (11). However, these are not reliable or generalizable strategies to determine parental *HBB* haplotype ratios.

Here, we addressed the issue of identifying the phase between SNPs and mutations by PCR amplifying the 2.2-kb region that includes the *HBB* gene in parents, sequencing this amplicon using Oxford Nanopore MinION single-molecule sequencing technology, and determining the parental *HBB* haplotypes using Soft Genetics’ NextGENe LR software. Having established the phase for the linked SNPs, we applied the observed sequence read ratios at these sites to infer which parental haplotype was transmitted to the fetus and thus predict with greater confidence the fetal genotype at the mutation site.

A recent NIPT paper (27) reported the Oxford Nanopore sequencing of a 10 kb and a 20 kb *HBB* amplicon. We focused on a shorter amplicon to ensure robust PCR amplification and minimize the potential for the formation of hybrid PCR sequences created by “template switching,” an artifact that is more likely with long amplicons (28). These artifacts could make the determination of haplotypes more difficult. Longer amplicons do allow more SNPs to be phased, but we showed that a 2.2 kb amplicon is sufficient for these purposes.

Our current protocol involves sequencing the plasma libraries, prepared by probe capture, on the Illumina MiSeq while sequencing the 2.2 kb *HBB* fragment PCR-amplified from the parental DNA on the Oxford Nanopore MinION. Though both our MiSeq and MinION library preparation protocols each take two days, sequencing via ONT takes 3.5 hours vs 1.5 days via MiSeq. Given this, library preparation and sequencing can be staggered so that parental *HBB* haplotypes are available to facilitate interpretation of the plasma read data as soon as the latter are generated.

A limitation of the current study is the absence of a probability estimate for the fetal genotype prediction. The development of such a metric is underway, and will be necessary for the establishment of probability thresholds and the definition of a “gray zone” indicating inconclusive results. Depending on how the probability thresholds are established, some of the current fetal genotype predictions deemed “correct” here may later be termed “inconclusive.”

In conclusion, where NIPT for autosomal recessive diseases may once have seemed very challenging, contemporary sequencing and informatic methods have made it a realistic clinical goal. The use of ISS of plasma reads increases the FF and the accuracy of fetal genotype prediction. Determining the parental beta-globin haplotypes further increases the predictive accuracy.
