# Correction to: Non-invasive prenatal testing of beta-hemoglobinopathies using next generation sequencing, *in-silico* sequence size selection, and haplotyping (*Croatian Medical Journal, https://doi.org/10.3325/cmj.2024.65.180*; published online June 2024).

**DOI:** 10.3325/cmj.2026.67.51

**Published:** 2026-02

**Authors:** Henry A. Erlich, Lily Ko, Jiyae Lee, Katrina Eaton, Cassandra D. Calloway, Ashutosh Lal, Reena Das, Manu Jamwal, Christian Lopez-Pena, Steven J. Mack

**Affiliations:** 1 UCSF Benioff Children’s Hospital Oakland Research Institute, Oakland, CA, USA; 2 Department of Pediatrics, University of California, San Francisco, Oakland, CA, USA; 3 Post-Graduate Institute of Medical Education and Research, Chandigarh, India

Since the publication of this article, the authors have determined that the Twist Bioscience probe panel used to accomplish the work was incorrectly identified as PanHeme panel TE-95715506. The actual panel used was PanHeme panel TE-93108959. This correction applies to the Methods/Probe Capture Enrichment section of the article. This was entirely a reporting error and does not affect the results or conclusions of the study.



